# Analysis of Waste Polymer Composition by a Simple Device for Raman Spectra Decomposition

**DOI:** 10.3390/polym18172085

**Published:** 2026-08-28

**Authors:** Jiří Militký, Karel Kupka, Dana Křemenáková, Mohanapriya Venkataraman

**Affiliations:** 1 Department of Material Engineering, Faculty of Textile Engineering, Technical University Liberec, 461 17 Liberec, Czech Republic; jiri.militky@tul.cz (J.M.); dana.kremenakova@tul.cz (D.K.); 2 TriloByte Statistical Software, 533 52 Pardubice, Czech Republic; kupka@trilobyte.cz

**Keywords:** quantification of fibrous mixtures, fibrous wastes, Raman mapping, microplastic identification, complex spectra decomposition

## Abstract

Today, the search for resources related to non-fossil raw materials that require less carbon-based energy consumption, use less water, and produce recyclable waste is prevailing. In the future, will this effort be replaced by resources sufficient to be produced in environmentally friendly ways? Resources will be circulated in a controlled manner, and materials will be sustainable. Most of the energy sources will be sustainable, derived from natural resources (sun, wind, waves, geothermal sources, etc.). The world will be managed by data, enabling resource sufficiency. Sustainable development is therefore a long-term strategy including economic, human (social), and environmental (material) resources. This strategy requires development in the complex identification and quantification of waste of different origins, including plastics and fibers. The identification of polymer complex mixtures and fibrous-blend waste, including microplastics, is a fundamental tool for effective environmental monitoring and comprehensive recycling management. Raman spectroscopy, combined with spectral unmixing techniques, provides a powerful tool for resolving overlapping spectral components and characterizing the composition of fibrous polymeric materials. The general goal of hyperspectral unmixing is to decompose an observed spectral mixture matrix into a set of pure component spectra and their respective portions (contributions or abundance) or ideally concentrations. This requires solving an inverse problem under physical and mathematical constraints. Principal component analysis (PCA), based on singular value decomposition (SVD), followed by independent component analysis (ICA) rotation, is used to reduce the number of components to a meaningful set of endmembers. The extracted endmembers should be statistically independent, nonnegative, and sum to one, because they represent real chemical components in the mixture. These requirements are fulfilled by constrained quadratic programming using the Newton linearization method. The RAMIX program, based on these procedures, has already been described, and its source code is available in another article written in Python. It is designed for the analysis of experimental Raman spectra of polymeric mixtures and for mapping waste fibrous blends. This program is used here for Raman spectral analysis of compressed textile samples composed of different staple fiber types. To evaluate Raman spectra, a simple, cost-effective, custom-built measurement system was created. This system allows mapping of fibrous mixtures by Raman spectra across a line or an area.

## 1. Introduction

Current society is characterized by excessive environmental pollution, rapid depletion of non-renewable material resources, high energy consumption, and the emergence of living-related troubles directly or indirectly linked to the frequency and magnitude of snowballing production/consumption cycles. Typically, the production of carbon dioxide (CO_2_) and other emissions is also growing, and the volume of waste placed in landfills is also increasing. The majority of improvements are still oriented toward isolated tasks rather than global solutions, and the prevailing competition among environmental, social, and economic aims [1].

Despite involving consumers in reducing fossil-based products, the generation of non-reusable waste and effective recycling management remain challenges, as identifying and quantifying product composition and corresponding waste streams are required primarily for recycling technologies.

One of the important parts of daily products is synthetic polymeric materials, which are widely used practically everywhere due to their technological advantages, unique material properties, and end-use versatility. Their advantages confirm the well-known DuPont slogan, “Better Living Through Chemistry.” These materials are characterized by low weight, sufficient mechanical and physicochemical properties, and durability during use. Synthetic polymer properties can be easily altered by the geometry of polymeric chains, including branching and crosslinking, as well as by supermolecular morphology, such as crystallinity and the orientation of morphological components [1].

A special class of polymers is fibers, which have a unique structural arrangement (primarily chain orientation) and distinct mechanical properties compared with polymers of the same chemical composition. Fibers are, in fact, long (typical range l = 10^−2^–10^−1^ m), thin (typical range d = 10^−6^–10^−4^ m) polymeric structures characterized by a so-called hierarchical fibrous structure. Special cases are filaments with a length equal to the product dimensions (called endless). In the case of natural fibers, the fibrous structure and geometry result from the conditions of fiber growth and are practically not influenced by humans. In the case of chemical and synthetic fibers, the fibrous structure and geometry result from their production processes and technological conditions, such as spinning, drawing, and heat setting. It is simple to change not only the length and thickness, but also the shape of the cross-section and longitudinal form (3D shape) [1]. In textile products, different kinds of fibers and blends (in the case of yarns) or mixtures of different kinds of yarns in fabrics often appear. Therefore, the identification, quantification, and mapping of the presence of different chemical fibrous components is essential for the characterization of textile fabrics and waste, including fibrous microplastics.

The major disadvantages of some synthetic fibrous structures include reliance on non-renewable resources, the production of long-lasting waste, and the presence of their degradation products as microplastics [1,2]. The textile industry is therefore a major contributor to environmental pollution, waste generation, and greenhouse gas emissions [3].

The classical techniques for the treatment of fibrous waste, such as landfilling, incineration, composting, and anaerobic digestion, often generate hazardous gaseous pollutants and are not ecologically acceptable. Incineration of textile waste is energetically convenient because its calorific value of 14,500 kJ/kg is comparable to brown coal. Textiles in landfills are releasing methane, CO_2_, and other toxic gases, depending on their chemical composition and the presence of chemicals on their surfaces. These techniques are typically downsizing and therefore not sustainable.

Upscaling methods, including depolymerization, are primarily used to produce recycled fibers from waste monomers. Typical examples are recycled PET fibers from waste PET bottles [1]. The basic routes for recycling different fibrous waste and fibrous mixtures (or blends) are discussed in depth, e.g., in [1]. Despite notable progress, the collection, sorting, and application of advanced or new technologies for textile recycling remains not widely practically harnessed [4,5].

For recycling purposes and avoiding microplastic danger, the identification of synthetic polymeric fibers’ chemical composition (type of polymer), which varies from simple polyolefins and their derivatives (polyethylene, polypropylene, PVC, etc.) to more complex materials (polyesters, polyamides, polyurethanes, etc.), is a key problem. Usually, polymeric waste is in the form of polymer mixtures, which complicate analysis and require separation into components (unmixing). There are many techniques for analyzing polymeric mixtures. Suitable ones are non-destructive (noncontact) methods based on small samples, such as FTIR or Raman spectroscopy, and their combination [6]. Despite their great advantages, including the simple identification of major typical groups in polymers, they have some limitations, including dependence on the morphological features of fibers [7], such as crystallinity and orientation, the presence of different dyestuffs (sample colors), and other chemicals in the mass or on the fibrous materials’ surface [5,8]. For mixtures of different polymers (as is typical of urban waste and textile-based microplastics), it will be challenging to use measured spectra to identify individual polymeric components [2,3,6,9].

The aim of this article is to demonstrate the utilization of a simple methodology for efficient decomposition of fibrous mixtures of Raman spectra into the components (endmembers) corresponding to individual components. The concepts of hyperspectral unmixing are mentioned, and the effective methodology, including data processing steps and constrained optimization, is discussed. This methodology served as the basis for the development of the RAMIX program described in [6]. This program is a tool for analyzing fibrous or polymeric Raman spectra obtained using a custom-made, simple, and cost-effective measurement system [6]. This system and software are validated by analyzing the Raman spectra of a real sample of polymeric fiber mixture.

## 2. Raman Spectroscopy

Spectroscopic techniques are based on the analysis of the absorption, reflection, and emission of molecules of materials when they come into contact with photons (generated standardly by lasers) [10,11]. The impact of photons causes the excitation of vibrational and rotational states of molecules, which are typical for different materials. Generally, the vibrational frequencies of a molecule depend on the nature of the motion, mass of the atoms, and type of chemical bonds [5,10].

Phonon scattering is mostly elastic (Raleigh or Mie), holding energy and color. The very small portion is inelastic scattering (Raman) with different energies and colors. Elastic scattering is typical for reflection, and inelastic scattering for emission [12,13].

For quantitative analysis of compounds containing organic and inorganic molecules, near-infrared (12,500–4000 cm^−1^, i.e., 0.8–2.5 μm), mid-infrared (4000–400 cm^−1^, i.e., 2.5–25 μm), and Raman spectroscopy (characterized by excitation laser wavelength in the visible or near-infrared region) are commonly used [10]. For chemical identification of waste polymeric particles, a spectrum of vibrational optical methods is used. ATR-FTIR spectroscopy is suitable for particles larger than 500 μm, micro-FTIR spectroscopy is applicable for particles in the size range of 20–500 μm, and Raman microspectroscopy is beneficial for particles larger than 1 μm.

Spectroscopic techniques, either alone or in combination with microscopes, are suitable for the identification and quantification of polymers and their mixtures [12]. The limitations often include the complicated identification of unknown substances and the difficult separation of multicomponent spectra.

Spectral measurements of polymer mixtures, rather than pure components, are more commonly used in practical applications [2,10,11] and for the analysis of fibrous mixtures [14]. It is quite difficult to interpret these combined spectra in terms of the spectra of individual components (polymers) and their amounts. Practically all synthetic fibers contain at least traces of rutile TiO_2_ used as a mating agent. Their presence creates one additional peak but does not change the rest of the Raman spectra [5]. Other agents containing Si-O bonds, such as surface-deposited silanes and glass particles, significantly alter the shape of IR spectra but have no influence on Raman spectra [5]. Very serious problems are caused by molecules with strong light absorption (such as black dyes or carbon black particles) and strongly fluorescent molecules (such as organic colorants, pigments, and dyes), which significantly alter spectra. These chemicals can contribute to lower spectral resolution and unsatisfactory reproducibility [5,13]. Also, some surfactants used in textile wet processing often exhibit strong Raman scattering and can be removed before spectral analysis by simple washing. On the other hand, Raman spectroscopy can be used to investigate the water-aggregation behavior of surfactants and ionic liquids [15]. In Raman spectra, very narrow, strong peaks (called spikes or cosmic rays) appear rarely due to interactions between high-energy particles and the detector. To avoid this random spectra corruption, it is simple to compare multiple measured spectra of the same materials.

Complex data from Raman spectroscopy are also processed using AI-based soft modeling techniques for quantitative fiber mixture analysis, which is necessary for waste classification and sorting [16]. Almost all effective soft modeling methods, such as neural networks, support vector machines, random forests, and decision trees, are used to improve sorting performance [16].

By detecting inelastically scattered light, Raman spectroscopy becomes an advanced analytical method in chemistry and materials science, allowing the identification and quantification of chemical species composition [17] and their metrological characteristics [7,15,18]. Raman spectroscopy is also an excellent tool for characterization of various materials’ defect density [19], but is not able to create spatial averaging in the case of non-uniform materials such as textiles. To eliminate this problem, Raman mapping was proposed to visualize and quantify spatial correlations between material characteristics and their non-homogeneity [19,20].

A spectral emission fingerprint is commonly produced by the distinctive Raman spectral peaks that each chemical component in a sample exhibits, corresponding to molecular vibrational modes. The typical Raman peaks of common fibrous polymers are shown in Table 1 [5,11,21].

A comprehensive evaluation of Raman spectra of common textile fibers, including their modifications and functionalization by nanoparticles, was published in ref. [5].

The titanium dioxide particles that make up real synthetic fibers often have a peak at 142 cm^−1^ (anatase form) [11]. They also contain additional side chemicals, such as finishing agents and dyestuffs, which might complicate their morphology.

Extracting pure spectral signatures (commonly referred to as “endmembers”) from complex hyperspectral images is a related issue that has driven substantial development in hyperspectral unmixing in remote sensing [22]. In such a case, the observed reflectance (or emission) of each pixel is represented by a combination of endmembers with matching abundance fractions. The fundamental concept is that a measured spectrum can be expressed as a linear combination of a finite number of constituent spectra, each properly weighted [23,24,25,26].

Decomposing an observed mixture Raman spectrum into distinct individual spectra (characterized individual polymers) and quantifying their relative proportions is a similar objective that can be achieved by adapting this framework, which was previously primarily applied to optical or infrared remote sensing data, to Raman spectra.

Adopting and modifying unmixing-based techniques for the breakdown of Raman spectra of polymers and waste mixtures is difficult, given their success in hyperspectral imaging [27,28,29,30].

## 3. Raman Spectra of Mixtures: Statistical Analysis

Experimental Raman spectra of mixtures and their components are a typical example of multivariate data forming matrices X with columns corresponding to the mixture and proposed components and rows corresponding to Raman intensities at different wavelengths. The basic characteristic of multidimensional data is its dimensionality (number of variables or possible components). Higher dimensionality brings problems with both data display and statistical analysis, and in the case of mixtures, it is a source of bad demixing. Some variables are noise (do not significantly affect the results) and can be excluded from the data (have no information value). Some variables are redundant because they are approximately linearly dependent or their relationships are given by physical constraints. In both cases, it is possible to replace the original variables with a reduced number of new or uncorrelated variables by dimensionality reduction [31].

There are three main problems when dealing with multivariate data:

1. Curse of dimensionality—the number of data needed to achieve the desired accuracy of multivariate estimates is an exponentially increasing function of the number of variables.
2. Empty subspace phenomenon—multidimensional data is concentrated in the peripheral regions of the parameter space.
3. Distance problem—the distance between objects is often strongly dependent on the strength of the coupling between the variables.

These problems require advanced techniques for accurate analysis [31].

Similar to univariate data, standard multivariate statistical analysis is performed here based on the parameters of location (mean vector) and dispersion (covariance or correlation matrix), finding outliers and verifying assumptions about the data and their distribution, especially normality.

Geometrically based multivariate data analysis replaces numbers from the very beginning with geometric objects (points, lines, axes, planes, figures) in a defined representation. It begins with the idea of data as a cluster of points in the Euclidean m-dimensional space E^m^. This is not only about the representation of data, but especially the quantitative investigation of mutual connections and dependences. The basis is the so-called Euclidean cluster, which is obviously the matrix **X** expressed as a cluster of points in the Euclidean space.

Most methods of multivariate statistical analysis allow the processing of linear multivariate models, where the response variables (mixture spectra) are considered as linear combinations of explanatory variables (polymeric component spectra), or the relationships between the variables are linear. In many cases, the normality of metric variables is also considered [31].

In particular, the primary objectives of polymer mixture analysis by Raman spectra are:

1. Decreasing the dimensionality of multivariate component spectra.
2. Extraction of a collection of significant individual spectra that signify different polymers (chemical substances) within the mixture.
3. Calculating the concentration (or contribution) of each compound (polymer) in every spectrum measured.
4. Guaranteeing that physically meaningful limitations are met, including the nonnegativity of concentrations and the necessity for concentrations to total one.

Raman intensities of the mixture samples are rows, and columns represent objects, namely, wavelengths, are shown. The issue arises from numerous variables (high dimensionality), and the inherent challenge is to diminish the dimensionality of the data matrix. The conventional approach to dimensionality reduction is principal component analysis (PCA), which involves a linear transformation (factorization) of the original coordinate system into a coordinate system of the principal components (latent variables) [31]. They are orthogonal (uncorrelated) and chosen to maximize the information gained from variations in Raman intensities. The significant principal components can be easily determined from the scree plot. This is a bar or point plot displaying the eigenvalues *L* (square roots of the singular values, representing a fraction of the overall variation in the original data) arranged by their size [31]. It allows rapid evaluation of the relative magnitudes of the eigenvalues from the slopes of lines in the scree plot. This plot is composed of a sharply decreasing first part (indicating important principal components) followed by a very slowly decreasing part (indicating less important principal components, not used for further calculations). Numerous writers suggest more quantitative, statistically motivated criteria and techniques for deciding how many principal components to keep (see [31]). The newly adjusted orthogonal coordinate system is turned in ways that maximize variability and reduce the distances of the objects from the principal components.

Concerning precision and dependability, the matrix S is decomposed directly via singular value decomposition (SVD). Following dimensionality reduction (via SVD), rotation (via ICA or comparable techniques), and the application of optimization constraints (nonnegativity, sum-to-one) are used to derive solutions with physical significance. The ultimate decomposition is chosen by minimizing reconstruction error and applying physical constraints, thereby ensuring that the identified endmembers reflect the actual chemical constituents of the mixture. Based on this analysis, a RAMIX program was developed in Python [6] and employed for all calculations realized here.

The unmixing ability of a RAMIX program was tested on twenty simulated random four-component mixtures of known elementary pure spectra of fibrous polymers (polyester, polyamide, cotton, and wool) with noise N(0, σ2) with σ = 0.05. The shape and proportionality of the reconstructed spectra and the location of peaks were in good agreement with the original spectra [6].

## 4. Experiment

**Figure 1:**
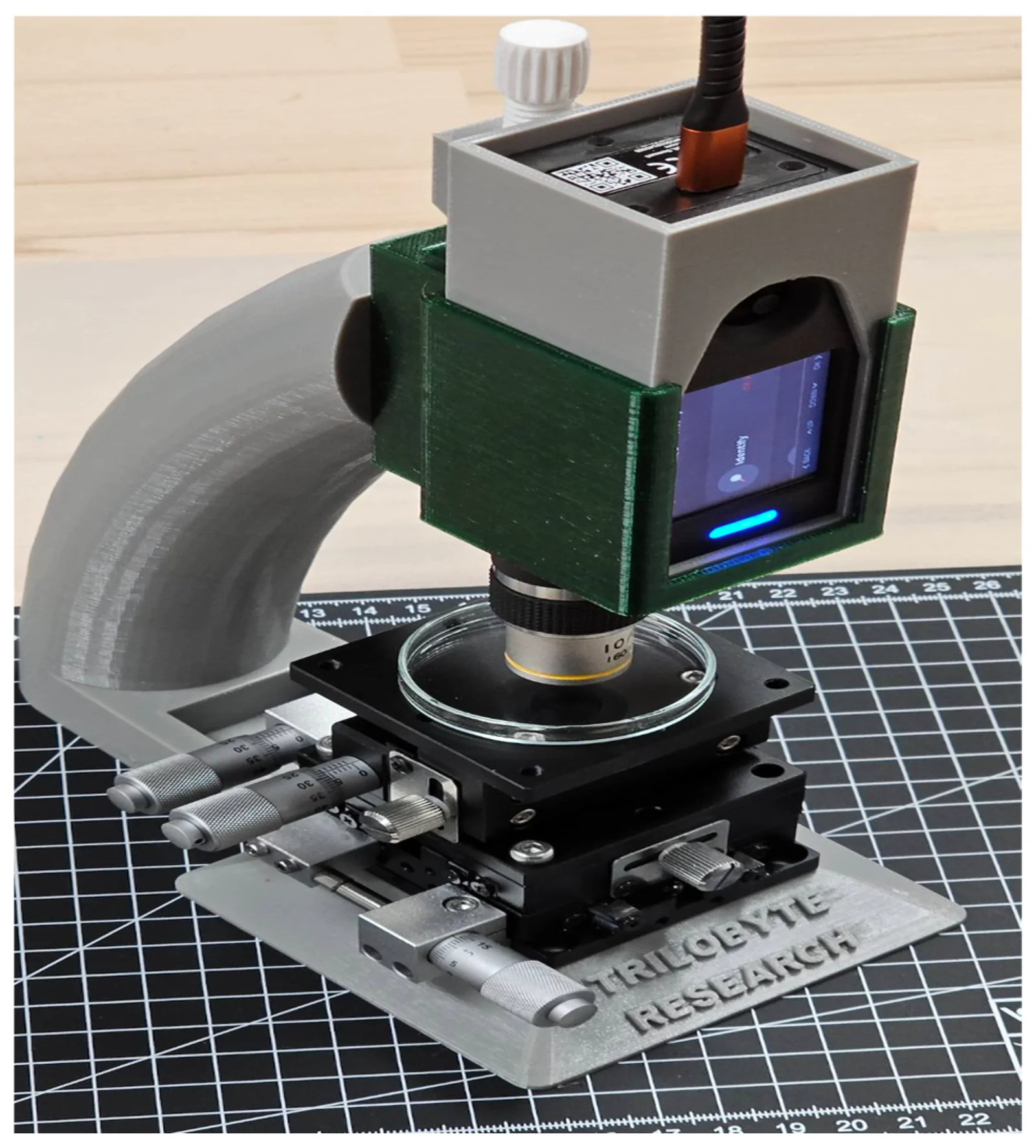
Measuring system composed of a mini Raman spectrometer (Lightnovo) on a stand with the XYZ calibrated table.

Analysis of fibrous polymer mixtures is increasingly important, and it has been a driving force for mapping the composition of fibrous mixtures across the line. A custom-made, low-cost measuring system was created. The main part of the system is a mini Raman spectrometer (LightNovo), working with a 785 nm excitation laser (see Figure 1—central black part). For precise selection of positions necessary for Raman spectra mapping across a line, a stand with micrometric XY shifts and coarse Z shift adjustment was built in cooperation with the company Trilobyte [32] (see Figure 1).

**Figure 2:**
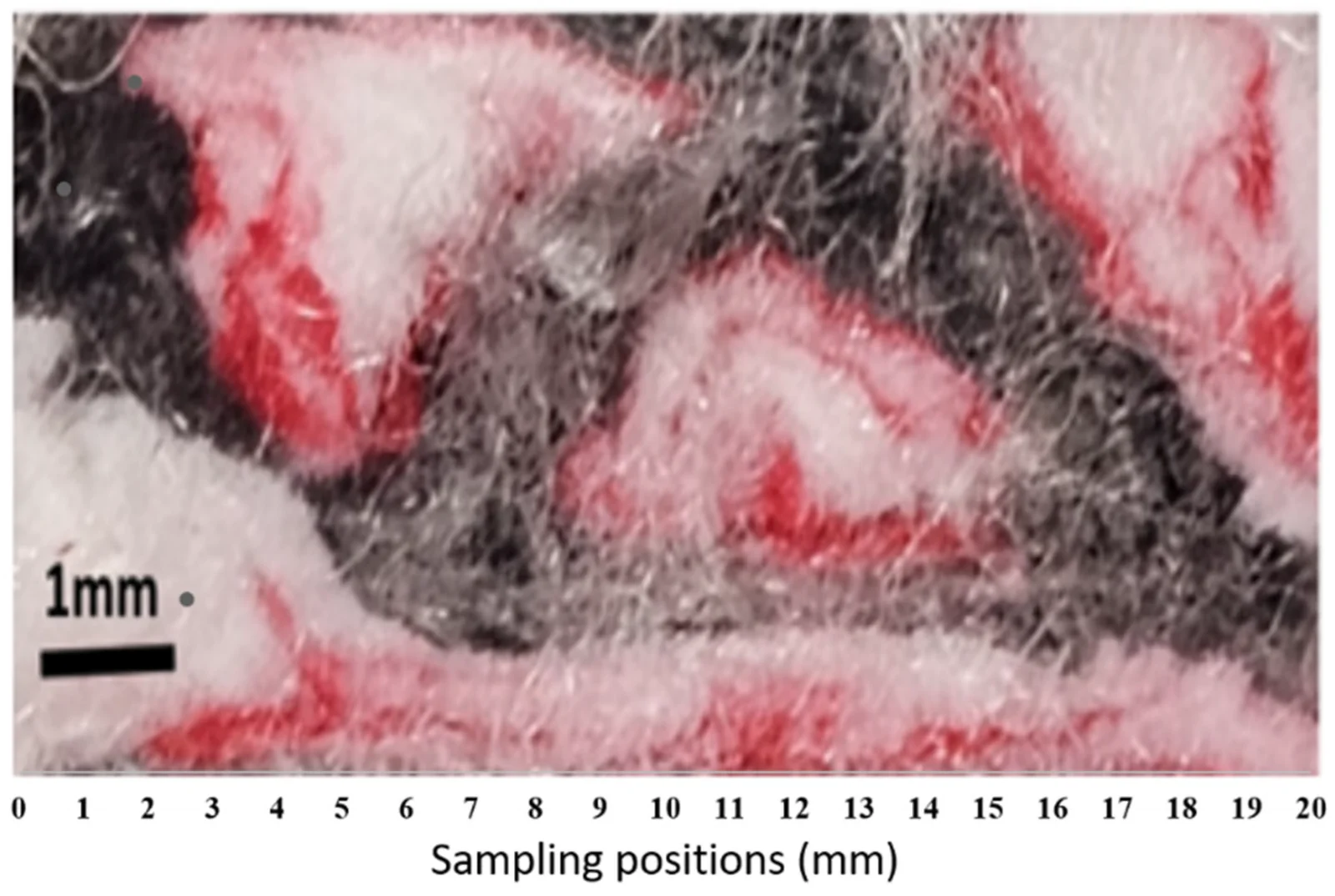
Textile waste mixture with selected sampling positions.

The proposed methodology was applied for the analysis of Raman spectra obtained from sample profiles of pre-processed waste samples from a compressed textile staple mixture (see Figure 2).

A series of Raman spectra was obtained over a sample line profile of length 20 mm (mapping across this line). The aim was to determine how the sample’s composition changed along the measured profile.

Sixty-four spectra measured at constant distances of 0.3125 mm in the whole sample profile were registered.

## 5. Results and Discussion

**Figure 3:**
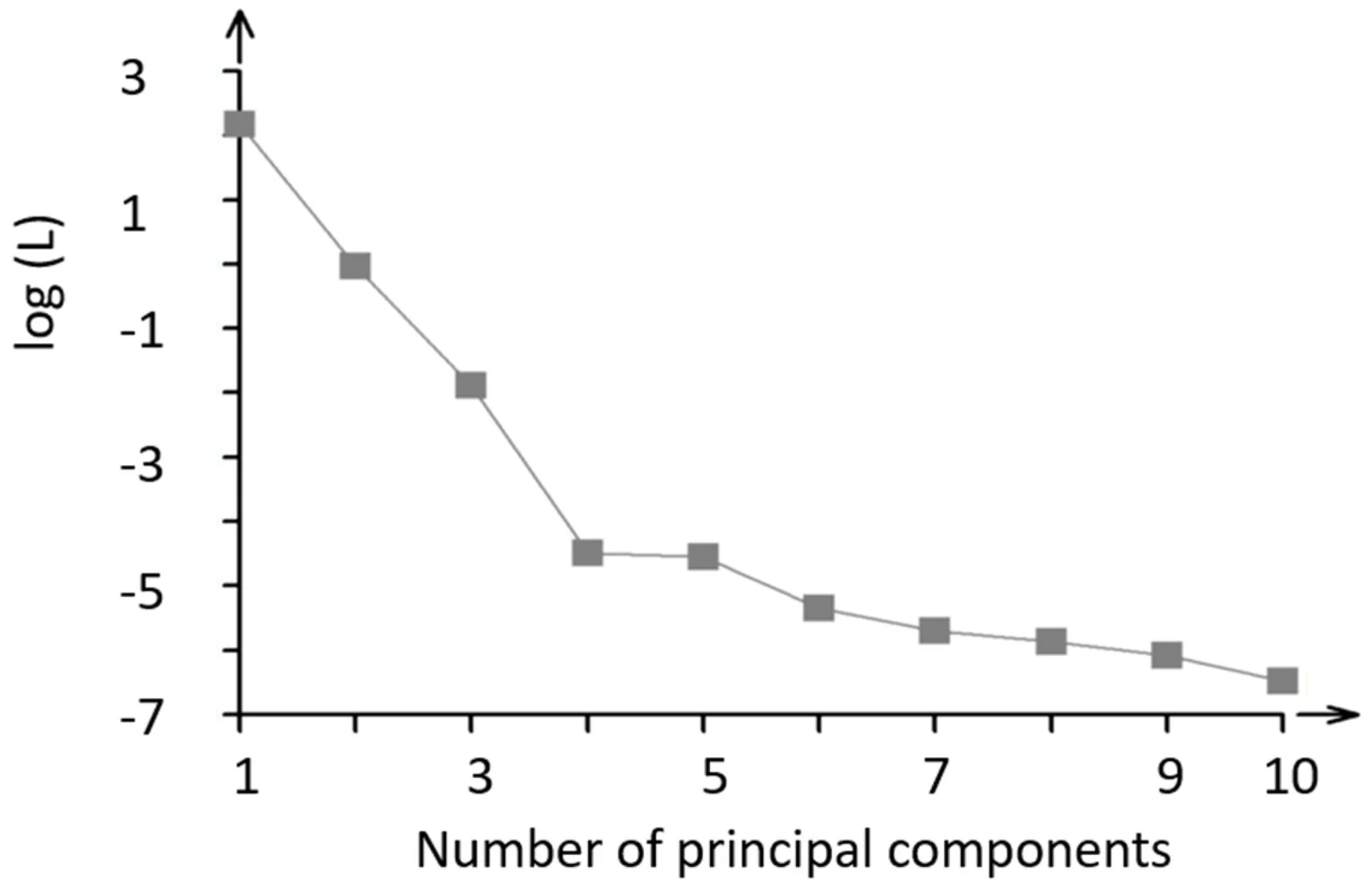
Scree plot for the determination of the number of important principal components.

The determined number of individual compounds from the PCA scree plot was equal to four (see scree plot in Figure 3 for decadic logarithms of individual eigenvalues *L*).

**Figure 4:**
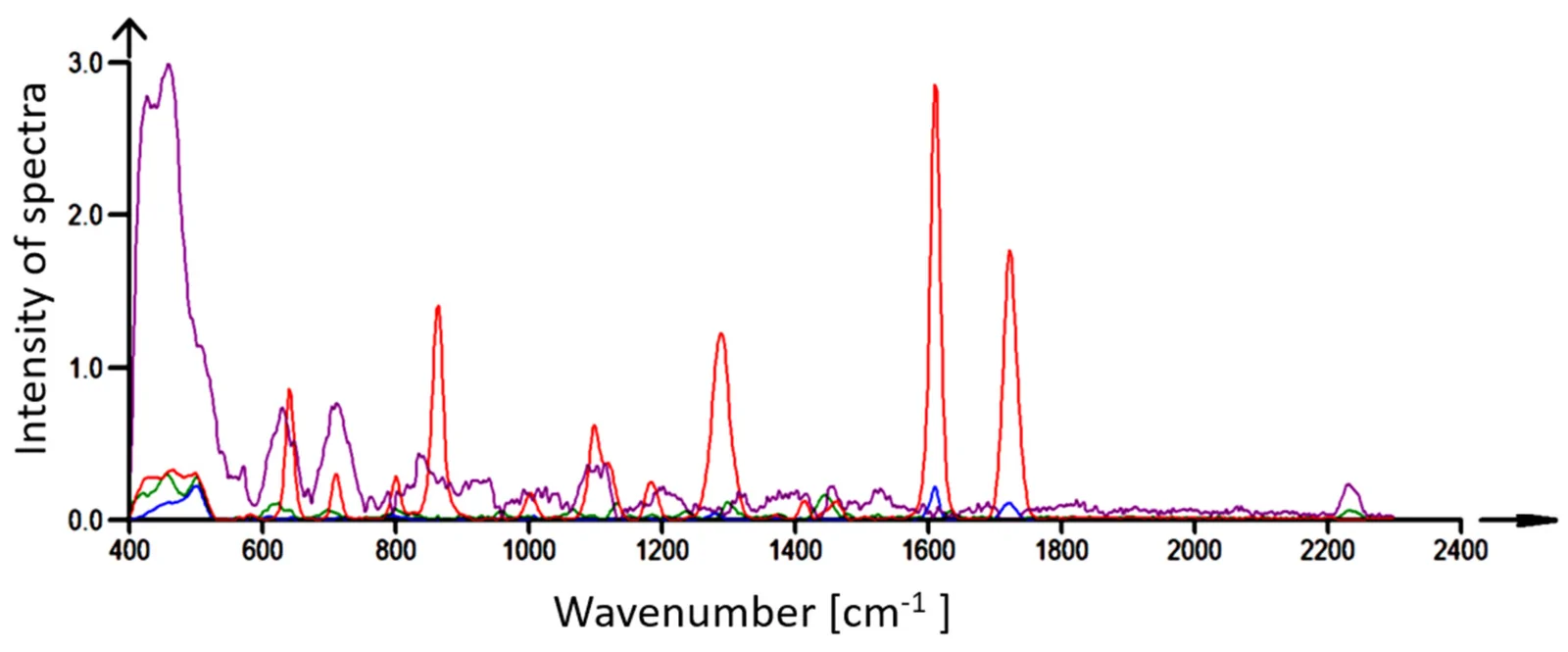
Textile waste mixture sample identified individual spectra.

The intensity *I* of reconstructed individual spectra for the individual fibrous components (fiber types) is shown in Figure 4. There are spectra of polyester (red), cotton (green), polyamide (violet), and wool (blue).

The RAMIX program was used for spectrum unmixing. Optimization procedure output: iteration time = 0.0042 s; total elapsed time = 4.9468 s. The objective function at minimum = 5.079684 indicates a low reconstruction error by using these four components only.

**Figure 5:**
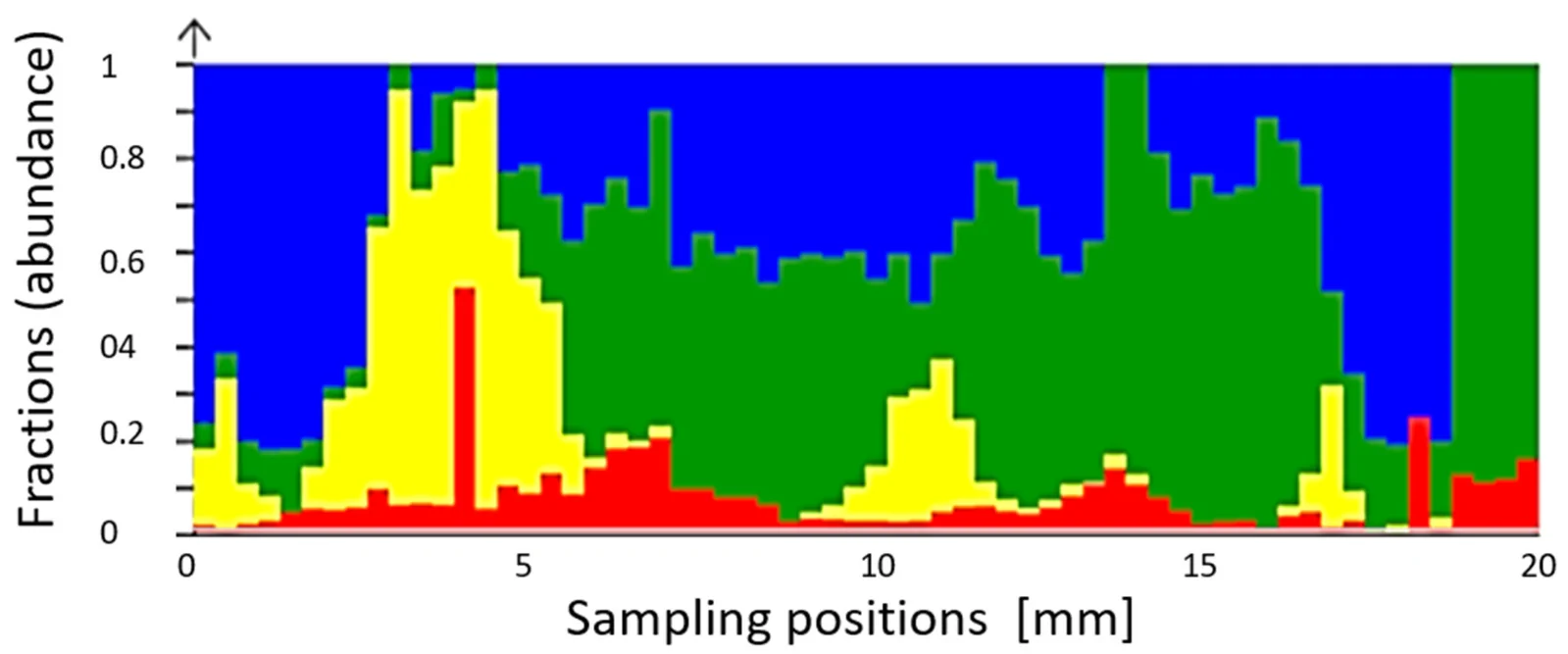
Textile waste mixture sample, reconstructed abundance profiles along the inspected 20 mm profile line in 64 equidistant sampling positions.

Figure 5 visualizes the determined abundances of the detected individual fibrous polymers in the profiles in selected sampling positions (measured at constant distances of 0.3125 mm) in the inspected 20 mm profile line.

It is clear that this methodology not only separates the mixture into components (individual fiber types) but also allows estimation of their proportions (concentrations) and identification of their distribution along positions specified by the profile line. This information can be used, e.g., to monitor sources of microplastics or to identify blends in textile waste.

## 6. Conclusions

Examining polymers has become increasingly significant across numerous scientific and industrial fields, particularly in environmental science, materials engineering, and recycling. Raman spectroscopy has proven to be a highly efficient, non-destructive technique for analyzing polymer materials, owing to its sensitivity to molecular vibrations and its ability to provide detailed spectral signatures. Discarded polymer materials, including old textiles, plastics, and particularly microplastics, often appear as complex mixtures with uncertain compositions. These materials exhibit spectral features associated with distinct polymer types, additives, and degradation byproducts. In these instances, spectral decomposition methods offer a valuable approach for identifying the primary constituent groups. Using spectral unmixing algorithms enables the separation of overlapping Raman signals and the extraction of distinct spectral signatures, leading to a more precise understanding of polymer mixture compositions. Consequently, Raman spectroscopy, when paired with sophisticated spectral unmixing techniques, serves as a powerful instrument for precisely identifying and delineating polymer components, especially in intricate, diverse samples. The developed measuring system for mapping Raman spectra can serve as a substitute for a pricier Raman spectroscopy system.

Upcoming studies will aim to enhance the use of Raman spectroscopy and microscopy for detecting and analyzing textile and plastic waste materials. An important aim will be to develop more effective spectral unmixing and analysis methods to better distinguish polymeric components in complex mixtures. Efforts will specifically focus on differentiating among widely used fibrous polymers, including polypropylene (PP), polyethylene terephthalate (PET), polyamides (PA 6 and PA6.6), acrylics, cotton (CO), and wools, as well as copolymers and mixtures, especially PET/CO. The utilization of machine learning-based spectral decomposition and sophisticated chemometric methods will be investigated to enhance spectral data analysis, especially for identifying degradation products and microplastic pollutants. To achieve higher resolution, it will be essential to develop a spectral database for these fibrous substances. Furthermore, the combination of Raman spectroscopy with additional imaging methods will be explored to enhance spatial resolution and to map diverse polymer waste samples. These advancements will enhance the accuracy and efficiency of polymer identification, aiding initiatives in recycling, pollution tracking, and sustainable material management.

## Figures and Tables

**Table 1:** Main Raman peaks of common fibrous polymers.

Type	Main Raman Peak [cm^−1^]
Cotton	331, 381, 438, 460, 1001, 1125, 1384, 1483, 2903
Wool	512, 881, 1002, 1245, 1338, 2880, 2931, 3062, 3302
PET	633, 857, 1096, 1289, 1615, 1727, 2970, 3093
PA 6	641, 1066, 1084, 1132, 1289, 1308, 1448, 1643, 2942
PA 6,6	957, 1059, 1134, 1238, 1302, 1446, 1643, 2882
PP	397, 808, 841, 973, 1152, 1329, 1360, 1459, 2884, 2956
PE	1062, 1129, 1295, 1440, 1460, 2846, 2881
PAN	142, 283, 1118, 1314 1466, 2246, 2916, 2967

## Data Availability

The original contributions presented in this study are included in the article. Further inquiries can be directed to the corresponding author.
